# Objective cognitive functioning in patients with stress-related disorders: a cross-sectional study using remote digital cognitive testing

**DOI:** 10.1186/s12888-023-05048-5

**Published:** 2023-08-07

**Authors:** Ludwig Franke Föyen, Victoria Sennerstam, Evelina Kontio, Mats Lekander, Erik Hedman-Lagerlöf, Elin Lindsäter

**Affiliations:** 1 Division of Psychology, Department of Clinical Neuroscience, Karolinska Institutet, Stockholm, Sweden; 2 Department of Psychology, Stress Research Institute, Stockholm University, Stockholm, Sweden; 3 Gustavsberg University Primary Care Center, Stockholm Health Care Services, Region Stockholm, Sweden; 4 Osher Center for Integrative Health, Department of Clinical Neuroscience, Karolinska Institutet, Stockholm, Sweden; 5 Center for Psychiatry Research, Department of Clinical Neuroscience, Karolinska Institutet, & Stockholm Health Care Services, Region Stockholm, Sweden

**Keywords:** Psychological stress, Cognitive impairment, Adjustment disorder, Exhaustion, Burnout, Digital technology, Cognitive test

## Abstract

**Background:**

Patients with stress-related mental disorders often report cognitive impairment, but studies investigating objective cognitive impairment in patients with stress-related disorders have produced inconsistent findings.

**Aim:**

The primary aim of this study was to investigate objective cognitive functioning in patients diagnosed with the stress-related disorders adjustment disorder or exhaustion disorder, compared to a healthy normative group. Secondary aims were to conduct subgroup analyses of cognitive functioning between the diagnostic groups and explore associations between self-reported symptoms and cognitive functioning.

**Methods:**

Cognitive test results on a digitally self-administered cognitive test battery from 266 patients (adjustment disorder, *n* = 131; exhaustion disorder, *n* = 135) were cross-sectionally compared with results from a healthy normative group (*N* = 184 to 692) using one-tailed *t*-tests. ANOVAs were conducted for subgroup analyses, and regression analyses for associations between self-reported symptoms and cognitive functioning. Effect sizes were calculated.

**Results:**

Patients performed significantly worse than the normative group on all measures with small to moderate effect sizes ranging from *d* = -.13 to -.57. Those diagnosed with exhaustion disorder performed worse than norms on more measures than did patients with adjustment disorder, but no significant differences between diagnostic groups were found on any measure. Self-reported memory impairment was weakly associated with one of two memory measures. No clear associations between self-reported burnout symptoms and objective cognitive functioning were found.

**Conclusions:**

This study adds to the literature indicative of small to moderate objective cognitive impairments in patients diagnosed with stress-related mental disorders. Further exploration into mechanisms of cognitive functioning in different populations is needed for development of theoretical models that may explain the weak correlation between self-reported symptoms and objective measures.

**Trial registration:**

ClinicalTrial.gov: NCT04797273. Trial registration date 15 March 2021. This study was also pre-registered on Open Science Framework (osf.io) with https://doi.org/10.17605/OSF.IO/TQXZV.

**Supplementary Information:**

The online version contains supplementary material available at 10.1186/s12888-023-05048-5.

## Background

Long-term exposure to non-traumatic life stressors in work and private life are associated with a range of psychiatric and somatic symptoms, functional impairment, and societal costs due to long term sick leave [1, 2]. The International Classification of Diseases (ICD) and The Diagnostic and Statistical Manual of Mental Disorders (DSM) dedicate sections to specify stress-related mental disorders. One of the stress-related mental disorders, adjustment disorder (AD), is conceptualized as a maladaptive reaction to non-traumatic life events that is characterized by excessive preoccupation of the stressor (e.g., constant worry, distressing thoughts, and rumination) and failure to adapt (as indicated by, e.g., disturbed sleep or difficulties concentrating) [3]. AD is one of the most used diagnostic constructs of all psychiatric diagnoses globally [3], and although many individuals remit spontaneously [4, 5], there are subpopulations of AD in which symptoms increase over time and may lead to more severe psychiatric disorders (e.g., [6]) and an increased risk for dementia [7]. The diagnosis exhaustion disorder (ED) was introduced to the Swedish version of the ICD (10^th^ edition) in 2005 [8] as a specification of the general diagnostic code F43.8 *Other reactions to severe stress*. Criteria for ED specify that symptoms develop in response to identifiable life stressors present for at least six months, resulting in persistent mental and physical fatigue for at least two weeks together with several other symptoms (e.g., reduced stamina, sensitivity to stress, and cognitive impairment (for diagnostic criteria, see Table S1, Supplementary material). ED shares similar clinical features with the internationally recognized construct of clinical burnout [9, 10]. AD and ED together have come to account for more than half of all cases of sickness absence due to psychiatric disorders in Sweden, and ED is responsible for more long-term sick leave episodes than any other disorder [11]. Importantly, even though ED is hypothesized to be a more chronic and functionally debilitating condition than AD, little is still known about the relationship and potential overlap between the diagnostic constructs. As such, a deeper understanding of the potential mechanisms involved in the functional disability, such as cognitive functioning, associated with AD and ED is highly warranted.

### Cognitive impairment in stress-related mental disorders

Patients with stress-related mental disorders and burnout symptoms often report cognitive impairments affecting attention and processing speed, executive functioning, and memory [12–14]. Although the association between self-reported cognitive complaints and stress-related mental disorders is well established in the literature, results of objective cognitive testing have been mixed. In the domain of attention, for example, some findings have indicated impairment in persons with ED and burnout symptoms [15–17] while others have not [18, 19]. There are also disparate findings within cognitive domains such as executive functioning [18, 19] and memory [15, 20]. Moreover, previous studies have indicated weak or no associations between self-reported cognitive impairment and cognitive test results [18, 21], implying that subjectively reported impairments may be influenced by other psychological factors, such as perfectionistic tendencies, heightened perception of threats, overinterpretation of cognitive failures, or negative self-perceptions [22]. In spite of the limited research available, sick leave recommendations for ED in Sweden state that reimbursement may be issued up to 12 months given persistent cognitive impairment [8], which far exceeds recommendations in comparison to other psychiatric disorders.

In a recently published meta-analysis by Gavelin et al. [13], cognitive functioning across a range of samples with stress-related conditions, such as ED and undifferentiated somatoform disorder (collectively referred to as “clinical burnout” in that study) were compared with that of healthy controls. The authors concluded that clinical burnout is associated with cognitive impairment in executive functioning, episodic and working memory as well as with attention and processing speed with small to moderate effect sizes. Importantly, no studies have to the best of our knowledge examined objective cognitive functioning in patients diagnosed with AD despite the common use of the diagnosis internationally [3] and studies of cognitive functioning in patients specifically diagnosed with ED are still few in number and have generally been underpowered [23]. Given the central role of cognitive impairment in the discourse surrounding stress-related mental disorders [13, 14, 23], and the impact cognitive impairment may have on questions regarding work ability and sick leave reimbursement, further investigation into this area is important.

### Aim of the study

The aim of the study was to examine cognitive functioning using a digitized self-administered cognitive testing platform in a large sample of patients diagnosed with AD or ED (*N* = 266) compared to a large normative group of healthy individuals (*N* = 184 to 692 depending on test). The primary hypothesis was that patients with stress-related mental disorders (AD or ED) as a group would perform worse in the cognitive domains of attention and processing speed, executive functioning, and memory compared to the normative group. To further the understanding of AD and ED as distinct diagnostic categories, exploratory analyses were conducted to compare each diagnostic group (AD/ED) against the normative group and to each other. Additionally, we explored the relationship between subjectively reported symptoms and results from the objective cognitive tests in the total clinical sample (AD and ED) by investigating the associations between (a) subjective memory impairment and the cognitive test results in the memory domain, and (b) subjectively reported symptoms of burnout and cognitive test results.

## Methods

### Study design

This study was part of a randomized controlled clinical trial (RCT) investigating the effect of internet-delivered treatments for patients diagnosed with AD or ED. The preregistration and full study protocol for the present study is available at Open Science Framework (osf.io) https://doi.org/10.17605/OSF.IO/7SAEU. Patients in the clinical trial were offered to complete a digital, self-administered cognitive test battery at baseline and at follow-ups. In the present study, using a cross-sectional design, the baseline cognitive test scores were compared with results from a healthy normative group. The study was approved by the Swedish Ethical Review Authority and all patients signed an informed digital consent before inclusion into the study.

### Patient recruitment and inclusion procedure

**Fig. 1:**
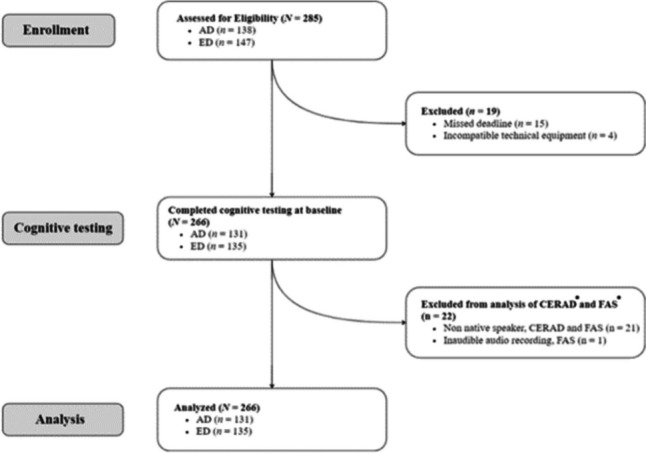
Consort diagram showing the inclusion/exclusion process. *Abbreviations*. AD, Adjustment disorder;  ED, Exhaustion disorder; CERAD, Consortium to Establish a Registry for Alzheimer’s Disease. *CERAD and FAS are cognitive tests used to assess memory and executive functioning and verbal fluency

Between the fall of 2021 and spring of 2022, 285 nationally recruited individuals were diagnosed with a primary diagnosis of AD (*n* = 138) or ED (*n* = 147) and included in the RCT in which cognitive functioning was one outcome. Of the included patients, 266 (AD, *n* = 131; ED, *n* = 135) completed cognitive testing at baseline. Figure 1 presents an overview of the inclusion and exclusion process.

Patient recruitment was carried out by information given to healthcare clinics, by newspaper ads and through social media. By self-referral on a study webpage, patients could sign a digital informed consent and respond to background demographic and clinical questions via BASS4, a secure online platform offered by the eHealth Core Facility at Karolinska Institutet, Stockholm. Patients were then assessed by a clinical psychologist using a structured diagnostic interview, the Mini International Neuropsychiatric Interview (MINI) [24] with additional diagnostic criteria for AD [25] and ED [8].

Inclusion criteria for the randomized controlled trial were: (1) primary diagnosis of AD or ED, (2) age 18–65 years, (3) regular access to a computer with internet access, and (4) ability to read and write in Swedish. Exclusion criteria were (1) drug use or addiction during the last 6 months, (2) current or previous bipolar disorder, (3) current risk of suicide, (4) changed psychopharmacological treatment in the past month, (5) ongoing other psychological treatment, and (6) cognitive behavioral therapy for a stress-related mental disorder within the past year. Specific exclusion criteria for the analysis of cognitive functioning were (1) non-native Swedish speaker for language sensitive tests (FAS, a test of verbal fluency and CERAD learning and delayed recall, see below) and (2) comments in the testing platform indicating an invalid test or measurement error (e.g., “*My computer froze during testing”)*. Of the 266 patients who completed cognitive testing at baseline, 21 patients were excluded from three of the cognitive tests (CERAD learning and delayed recall, FAS) because they were non-native Swedish speakers. One patient was also excluded because of an inaudible audio recording (FAS). No patients were excluded based on patient comments in the testing platform.

### Healthy normative comparison group

Cognitive test results from a healthy normative group were provided by the testing platform Mindmore [26, 27]. Norm group data for three of the subtests, CERAD (*n* = 186), Corsi Forward (*n* = 692) and FAS (*n* = 184) were collected between 2018 to 2020 on-site in Mindmore’s office in Stockholm using Windows tablets with healthy Swedish speaking participants. Participants were recruited from a website specialized in research participant recruitment, in social media, by posters, during retirement association meetings and by word of mouth. For full information on exclusion/inclusion criteria, recruitment and testing procedure as well as participant characteristics for the healthy normative group, see van den Hurk et al. [26].

For SDMT (*n* = 290) and Stroop (*n* = 321) norm group data were collected between 2021 and 2022. Participants were recruited by a website specialized in research participant recruitment, through posters, by social media, and by word of mouth. Participants completed the tests using a personal computer at home. Exclusion criteria were the following: (1) non fluent level of Swedish (2) previous experience with the testing platform, (3) impaired cognitive functioning as indicated by a health form, (4) an active central nervous system disease or a psychiatric disorder, (5) prior history of a disorder, including substance or alcohol use disorder that could potentially affect cognition, (6) colorblindness (for Stroop), (7) Hospital Anxiety and Depression Scale-D (HADS-D) score ≥ 11, (8) Hospital Anxiety and Depression Scale-A (HADS-A) score ≥ 11, (9) Karolinska Exhaustion Disorder Scale ≥ 19, and (10) Attention Deficit Hyperactivity Disorder with or without medication.

### Measurements

#### Cognitive tests

All cognitive tests were provided by Mindmore AB and administered digitally for patients to complete via their private computers. The cognitive test battery consisted of five tests with a total of seven measures used to assess three overarching domains: attention and processing speed, executive functioning, and memory.

##### Attention and processing speed

Symbol Digit Modality Test (SDMT) [28] was used to assess attention and processing speed, and visual detection. A key with nine different symbols and matching numbers is shown on the upper part of the display. At the center, one of these nine symbols is shown, and the task is to choose the corresponding number at the bottom of the display in a 3 × 3 matrix using the key as guidance. The test score is the number of correct entries in 90 s.

##### Executive functioning

FAS [29] was used to measure spontaneous verbal fluency, switching and inhibition within the domain of executive functioning. The participant is tasked with producing words beginning with a certain letter of the alphabet (F, A, and S). Names, numbers, or repeated words are not allowed. Test score is the number of correct words beginning with the letter.

Stroop test [30, 31] was used to assess executive functioning, inhibition, selective attention, updating as well as processing speed. The test has two parts: First, twenty color words are presented (green, yellow, blue, or red) and they are colored congruent to their meaning (e.g., the word red colored in red). In the bottom part of the display the color words are displayed on four buttons. The task is to, as quickly and thoroughly as possible, click the button corresponding to the color word presented. Second, twenty color words are again presented but displayed in an incongruent color (e.g., the word red colored in green). The task of the participant is to click the button containing the color of the word as quickly as possible. Test score is calculated as an index (number of correct answers in part two/average time in seconds from part two) and for interference (average time in part one – average time in part one).

##### Memory

CERAD Word List Learning Test was used to assess verbal learning and episodic memory. It was originally developed for use with Alzheimer’s disease [32] but is similar to other word-list tasks used in populations with stress-related mental disorders (e.g. [18, 33]). In the learning part of the test, a word list containing ten words is presented over three trials and the task after every trial is to recall the words from the list. For every presentation the order is mixed. In the delayed recall part of the test (trial 4) that occurs after 5–10 min, the participant is asked to recall the words. Test score for the learning time is number of correct words in trial 1–3 (maximum of 30 correct answers), and in the delayed recall part, number of correct words in trial 4 (maximum of 10 correct answers).

Corsi block-tapping test forward was used to measure visual attention, short term visuospatial and working memory [34]. It contains two parts, forwards and backwards, but the test battery only included the first part of the test because of time-restrictions. Nine blocks are displayed, and the testing platform starts by lighting up a sequence of blocks. The task is to repeat the sequence of blocks that the platform has displayed. The task starts out with two blocks, but for each subsequent round the difficulty increases by adding a longer sequence of blocks until the participant enters the incorrect sequence twice at the same number of blocks. The test score is the maximum number of correct repeated blocks.

### Self-report questionnaires

The self-report questionnaires were administered online after inclusion in the study and before randomization. A revised version of the six-item questionnaire of Everyday Memory Problems (6-QEMP) specifically adapted for patients with stress-related mental disorders was used to measure subjective memory impairment [21]. It included statements such as “How do you think your memory functions now compared to before you developed stress-related mental health problems?”. The Swedish version used can be found in the Supplementary material.

The Shirom Melamed Burnout Questionnaire (SMBQ-18) [35, 36] was used to assess self-rated burnout. It aims to measure three components of burnout, i.e., *Physical fatigue, Cognitive weariness,* and *Listlessness*. Each item is rated using a 7-point scale ranging from 1 (Never or almost never) to 7 (Always or almost always). For each component and the total scale, the score is averaged by dividing to the number of items in each domain. A total score of 4.4 has been suggested to separate individuals with burnout from healthy individuals [35].

### Testing procedure

After inclusion, patients were sent an email with a link to access the cognitive tests.

Patients could take the test at any time of the day in their own home using their own hardware and were encouraged to take the test in a quiet and calm environment. As the patient clicked the test link, they received practical information regarding the testing, such as time of testing (≈20 min) with no breaks allowed. Data was collected on what input method (mouse, touchpad, touchscreen) the patient used since this can affect time-based tests and is used as a predictor for the normative regression models. Testing then proceeded with each sub-test in the following order: Corsi blocking tapping test, CERAD learning, Stroop, SDMT, CERAD recall, and FAS.

After testing, the patient was asked if there were any disturbances or other difficulties during the test. The testing platform automatically calculated and provided raw results for SDMT, Stroop test, and Corsi. For CERAD and FAS, each audio recording was manually reviewed and corrected by Mindmore staff.

### Statistical analysis

Following inclusion/exclusion of cognitive test results based on aforementioned criteria, data was standardized according to multiple linear regression models resulting in *z*-scores indicating patient results in standard deviations above or below expected score. The models were provided by the testing platform Mindmore and give an expected result for each patient given their age, sex, education, and input method*.* These models were calculated based on the healthy normative comparison group previously described. For a full review of the multiple linear regression models used, and how they were calculated, see [26, 27].

To test for normality of the standardized patient scores, data was visually inspected and a subsequent Shapiro–Wilk test was conducted. For our primary hypothesis, if patient data was acceptably normally distributed, an independent one sample, one-tailed *t*-test was used to test for differences between the expected and the actual score. Effect sizes as well as confidence intervals were calculated using Cohen’s *d*. For non-normal data, a one-sample Wilcoxon signed-rank test was used. Effect sizes were calculated using matched rank biserial correlation.

For the first exploratory analysis, to compare the diagnostic groups separately with the normative group, a virtual control was generated for each patient by taking the expected cognitive test results given the model, and its standard deviation, and generating a result from a normal distribution with the expected score as mean. In addition, for CERAD learning, recall, and Corsi, generated scores that were above the maximum limit for the test (i.e., 30 words for CERAD learning, 10 words for recall, and 9 blocks for Corsi) were adjusted to account for the inherent ceiling effect of the test. Creating a virtual normative group was done as an alternative to conducting multiple one-sample *t*-tests (7 × 3) for each test and diagnostic group (an example of how this was done is provided in the Supplementary material). Using this virtual normative group, the diagnostic categories (AD and ED respectively) were compared against the norms and to each other using a one-way ANOVA for each cognitive test. If the ANOVA was significant, a post-hoc Tukey HSD was used for pairwise comparisons between the groups (AD vs ED; AD vs normative group; ED vs normative group).

For the second exploratory analysis, the test results in the memory domain (CERAD learning and delayed recall as well as Corsi) were correlated to the subjective memory impairments using the sum-score of 6-QEMP. Additionally, each cognitive test was correlated to the self-rated burnout symptoms using the sum-scores of the SMBQ-18. The size of the sample was determined by a power analysis for the main randomized trial investigating treatment effects, as such, no power analysis for the present study was conducted. No Bonferroni corrections for multiple comparisons were made for our primary or exploratory analyses. However, post-hoc test showed that when using the Benjamini–Hochberg procedure applying a false discovery rate of 5% for the primary analyses, all significant tests (presented in Table 2) remained significant which suggests that false positives was not a major issue.

## Results

### Patient characteristics

Table 1 reports patient characteristics for the total group of patients with stress-related disorders, as well as for respective diagnostic group (AD and ED). As can be seen, the diagnostic groups differed significantly on most demographic and clinical factors. Compared with AD patients, for example, ED patients reported longer symptom duration, were on sick leave to a greater extent, had a higher level of comorbidity, and reported more memory impairment and burnout symptoms.

**Table 1:** Baseline characteristics of the total sample with stress-related disorders as well as for the diagnostic subgroups adjustment disorder (AD) and exhaustion disorder (ED)

	**Total *(N* = 266)**	**AD (*n* = 131)**	**ED (*n* = 135)**	***p*-value**
**Sex, Women *n* (%)**	239 (89.85)	114 (87.02)	125 (92.59)	.133
**Age, year**				
*M* (*SD*)	44.55 (9.29)	42.97 (9.77)	46.09 (8.59)	**.006**
Minimum—maximum	25–66	25–66	25–63	
**Highest education, *n* (%)**				**.002**
College/university, ≥ 3 years	178 (67)	101 (77)	77 (57)	**.001**
College/university, < 3 years	46 (17)	15 (11)	31 (23)	**.013**
Secondary school, 2–3 years	42 (15.79)	15 (11)	27 (20)	.056
**Employment status, *n* (%)**				** < .001**
Full time	144 (54.14)	98 (74.81)	46 (34.07)	** < .001**
Part time (25—90%)	63 (23.68)	19 (14.5)	44 (32.59)	**.001**
Other^a^	49 (18)	12 (9)	37 (27)	** < .001**
Unemployed	10 (3.76)	2 (1.53)	8 (5.93)	.059
**Sick leave, *n* (%)**	57 (21.43)	4 (3.05)	53 (39.26)	** < .001**
**Self-reported symptom duration (years)**				
*M* (*SD*)	2.19 (1.89)	1.85 (1.83)	2.52 (1.9)	**.004**
Median	1.67	1.33	2	
Minimum—maximum	.25–10	.25–10	.25–10	
**Comorbid psychiatric diagnosis *n* (%)**	62 (37.78)	11 (8.4)	51 (23.31)	** < .001**
Anxiety disorders	25 (9.4)	6 (4.58)	19 (14.07)	**.015**
Depression	28 (10.53)	3 (2.29)	25 (18.52)	** < .001**
Other^b^	9 (3.38)	2 (1.53)	7 (5.19)	.19
**Self-reported neuropsychiatric disorders**	17 (6.39)	6 (4.58)	11 (8.15)	.348
**Medication, *n* (%)**	120 (45)	36 (27)	84 (62)	** < .001**
Anxiolytics	14 (5.26)	4 (3.05)	10 (7.41)	.112
Sleeping medication	49 (18.42)	16 (12.21)	33 (24.44)	**.01**
Antidepressants	53 (19.92)	16 (12.21)	37 (27.41)	**.002**
Pain medication	4 (1.5)	0 (0)	4 (2.96)	.139
**Swedish native speaker, *n* (%)**	245 (92.11)	117 (89.31)	128 (94.81)	.096
**6-QEMP**				
*M* (*SD*)	14.65 (3.95)	12.95 (3.53)	16.3 (3.65)	** < .001**
Minimum—maximum	5—24	5—22	8—24	
**SMBQ-18**				
*M* (*SD*)	5.21 (0.89)	4.82 (.84)	5.58 (.79)	** < .001**
Minimum—maximum	2—7	2—6.56	3.5—7	

*Abbreviations*. *6-QEMP,* Six-item questionnaire of Everyday Memory Problems; *SMBQ,* Shirom Melamed Burnout Questionnaire 18-items

^a^Student, retired, parental leave or disability pension

^b^Insomnia, Bulimia nervosa

Of the total number of patients (*N* = 266), the majority (*n* = 174, 65.41%) completed the test between 08:00 and 17:00. A considerable number of patients (*n* = 77, 28.95%) completed the test between 17:00 and 22:00, while a smaller group of patients (*n* = 15, 5.64%) completed the test between 22:00 and 08:00.

### Differences between total sample of patients and healthy normative group

As shown in Table 2, the clinical sample had lower scores on all cognitive tests as compared to norms, thus indicating lower functioning in the domains of attention and processing speed, executive functioning, and memory.

**Table 2:** Cognitive test results for patients with stress-related mental disorders as compared to norms

Cognitive test	*Mean*^*1*^ (*SD*)	Test statistic^2^	*df*	*p*-value^3^	Effect size^2^	95% CI
**Attention and processing speed**						
SDMT	-.44 (.92)	-7.77	265	** < .001**	-.48	[-.60, -.35]
**Executive functions**						
FAS	-.11 (.82)	-2.03	243	**.022**	-.13	[-.26, 0]
Stroop index	-.62 (1.13)	7699	265	** < .001**	-.57	[-.65, -.47]
Stroop interference	-.28 (1.45)	15224	265	**.022**	-.14	[-.28, -.01]
**Memory**						
CERAD learning	-.33 (1.13)	10390	244	** < .001**	-.31	[-.44, -.18]
CERAD recall	-.5 (.92)	6983	244	** < .001**	-.54	[-.63, -.47]
Corsi	-.22 (.95)	12174	265	** < .001**	-.31	[-.43, -.19]

*Abbreviations. CERAD,* Consortium to Establish a Registry for Alzheimer’s Disease; *SDMT,* Symbol Digit Matching Task

^1^*Mean* signifies the mean of *z*-scores in the patient group

^2^For SDMT and FAS, the Test statistic is the *t*-statistic, and the effect size is given by Cohen's *d*. For Stroop index and interference, CERAD learning and recall and Corsi, Test statistic is the value of the *W*-statistic and effect size is given by the matched rank biserial correlation

^3^For the Student *t-test*. the alternative hypothesis specifies that the mean is less than 0. For the Wilcoxon test the alternative hypothesis specifies that the median is less than 0

A post-hoc sensitivity analysis with outliers ± 3 *z*-scores removed found impaired cognitive functioning in patients for all tests except for Stroop interference. For more information, see the Supplementary material and Table S2.

### Differences between diagnostic groups

One-way ANOVAs were used to test for group differences in *z-*scores between the patients diagnosed with AD, ED, and the virtual controls. The ANOVAs were significant for SDMT *F*(2, 529) = 1.26, *p* < 0.001, η2 = 0.04, Stroop index *F*(2, 529) = 26.91, *p* < 0.001, η2 = 0.09, and Stroop interference *F*(2, 529) = 3.97, *p* < 0.001, η2 = 0.02, CERAD learning *F*(2, 487) = 5.49, *p* < 0.001, η2 = 0.02, CERAD recall *F*(2, 487) = 16.82, *p* < 0.001, η2 = 0.06, and for Corsi *F*(2, 529) = 3.8, *p* = 0.023, η2 = 0.01. The only non-significant ANOVA was FAS *F*(2, 486) = 0.86, *p* = 0.424, η2 = 0. An overview of all significant ANOVAs can be found in Table S3, Supplementary material.

Table 3 presents post-hoc analysis using Tukey HSD. For SDMT, Stroop index and interference, and CERAD learning and recall, pairwise comparisons between patients with ED and the virtual control group showed a statistically significant difference with ED patients performing worse than controls. Comparisons between patients with AD and the virtual control group showed a difference for SDMT, Stroop index, and CERAD recall, where patients with AD performed worse than controls. When comparing diagnostic groups to each other, there were no statistically significant differences in cognitive test performance. For Corsi, used to measure visual attention, short term visuospatial and working memory, there were no significant subgroup differences.

**Table 3:** Pairwise comparisons of cognitive test results between adjustment disorder (AD), exhaustion disorder (ED), and normative group (NG) using post-hoc Tukey HSD

Cognitive test	Comparison		Mean difference^1^	95% CI	*p*-value^2^
Attention and processing speed					
SDMT	AD	ED	-.20	[-.48, .07]	.197
		NG	.27	[.02, .51]	**.027**
	ED	NG	.47	[.23, .71]	**< .001**
Executive functions					
Stroop index	AD	ED	-.27	[-.57, .03]	.093
		NG	.50	[.24, .77]	** < .001**
	ED	NG	.77	[.51, 1.04]	**< .001**
Stroop interference	AD	ED	-.30	[-.67, .07]	.132
		NG	.07	[-.24, .39]	.848
	ED	NG	.37	[.06, .69]	**.015**
Memory					
CERAD learning	AD	ED	-.14	[-.46, .18]	.549
		NG	.23	[-.05, .51]	.137
	ED	NG	.37	[.1, .65]	**< .001**
CERAD recall	AD	ED	-.09	[-.35, .17]	.684
		NG	.40	[.17, .63]	**< .001**
	ED	NG	.49	[.27, .72]	**< .001**
Corsi	AD	ED	-.01	[-.3, .28]	.998
		NG	.24	[-.02, .49]	.072
	ED	NG	.25	[-.01, .5]	.057

*Abbreviations. CERAD,* Consortium to Establish a Registry for Alzheimer’s Disease; *SDMT,* Symbol Digit Matching Task

^1^Mean difference indicates difference in *z*-score with a positive value meaning a higher score in the right column

^2^*p-value* adjusted for comparing a family of three for each cognitive test

### Correlations between subjective memory impairments, burnout symptoms, and cognitive test scores

In the clinical sample (AD and ED), CERAD learning was negatively correlated with subjective memory impairment as assessed with the 6-QEMP, *r*(243) = -0.21, *p* < 0.001, and so was CERAD recall *r*(243) = -0.14, *p* = 0.029. No relationship between Corsi and subjective memory impairment was found. Additionally, correlations were computed between SMBQ-18, which measures symptoms of burnout, and all cognitive tests. Out of seven measures, only FAS was significant, *r*(242) = -0.19, *p* = 0.003, with higher SMBQ-18 scores being associated with worse performance on FAS. For a heatmap showing the relationships between cognitive test scores and subjective symptom ratings, see Table S1, Supplementary material.

## Discussion

This study investigated cognitive functioning in patients diagnosed with stress-related mental disorders operationalized as adjustment disorder (AD) and exhaustion disorder (ED). In line with our hypothesis, results showed impairments in all cognitive domains (attention and processing speed, executive functioning, and memory) compared to the normative group, with small to moderate effect-sizes. The impairments were most pronounced in the domains of executive functioning (Stroop index) and memory (CERAD recall). Explorative subgroup analyses indicated that patients diagnosed with ED performed worse than healthy controls on five of seven measures (all except FAS and Corsi), while patients with AD performed worse than healthy controls on three of seven measures (SDMT, Stroop Index, and CERAD recall). When comparing diagnostic groups to each other, no significant differences in cognitive functioning were however found. Verbal learning and episodic memory (CERAD), but not short term visuospatial and working memory (Corsi), was associated with self-reported memory impairment. There were no clear associations between severity of burnout symptoms and objective cognitive functioning.

The main findings from the present study are largely consistent with results from a recent meta-analysis demonstrating small to moderate impairment in several cognitive domains in patients with stress-related mental conditions [13]. The moderate impairments found in the domain of memory and executive functioning in the present study were however larger than those of the meta-analysis, and in contrast to the meta-analysis, the current study also found small impairments in visuospatial ability as measured by Corsi. The meta-analysis conversely found a moderate effect size for fluency, whereas the present study found a small effect. Of note, the meta-analysis included a broader sample of diagnostic categories as well as burnout (that is often defined somewhat differently in different studies), which could likely explain some of the differences in outcomes.

Across individual studies specifically investigating cognitive functioning in patients diagnosed with ED, inconsistent findings are common. For example, in the domain of attention and processing speed, Ellbin et al. [15] found that patients with ED performed worse than controls using SDMT, while Jonsdottir et al. [16] did not when using a comparable digit symbol matching task. In the domain of executive functioning, Ellbin et al. [15] found that patients with ED performed worse than controls. However, other studies have not detected differences using the same measure [16, 18]. Concerning memory, Jonsdottir et al. [16] found impairments in patients with ED for delayed recall using a logical memory task, but not on immediate recall. Nelson et al. [18] reported heterogenous results in working memory with ED patients performing worse in one of three tests, and no difference in episodic memory. Additionally, Sandström et al. [33] found no differences between patients with ED and controls using a word list task similar to CERAD, but saw worse performance in patients using a visuospatial memory task. Several factors may account for the inconsistencies in results, including small sample sizes in previous studies, differing cognitive tests, varying inclusion and exclusion criteria, and differences in patient and comparison group populations regarding, for example, recruitment strategy. These and other shortcomings have also been pointed out in the meta-analysis by Gavelin et al. [13]. Moreover, to the best of our knowledge, no previous studies have investigated cognitive functioning in patients diagnosed with adjustment disorder despite its common use in clinical settings [3].

The present study differs from previous studies of cognitive functioning in patients with ED in several ways. First, the sample size was significantly larger than in most previous studies, likely enabling us to detect smaller effects. Further, in the present study, patients were able to complete the test in the comfort of their own homes which might have contributed to a more ecologically valid picture of cognitive functioning than testing done in a formal clinical setting. Indeed, results by Gavelin et al. [37] indicated that patients with ED showed an increase in neuronal resources compared with healthy controls during a structured on-site testing situation, which authors argued might be an indication of patients compensating for actual cognitive deficiencies. This being said, it is important to acknowledge that the testing procedure used in the current study might have introduced bias, such as a potential decrease in motivation among participants and less control over potential external disturbances during testing. Clearly, further research is needed to investigate the impact of specific testing procedures and context on the impact on cognitive performance.

To probe the cognitive profiles of patients with AD and ED respectively, we conducted several exploratory analyses. Patients with ED performed significantly worse than the virtual control group on more cognitive tests than did patients with AD, possibly suggesting more wide-spread cognitive difficulties in the ED population. This is perhaps not surprising given the demographic and clinical data indicative of significantly longer symptom duration, more psychiatric comorbidity, less full-time work, and higher frequency of medication in the ED sample compared to AD. However, when the diagnostic groups were directly compared there were no significant differences between their cognitive performance on any test, even though there were trends towards lower performance in the ED group. A recently published scoping review of all published studies on ED indicated that the diagnostic construct is heterogeneous and that there is little empirical evidence to support the specificity of the diagnosis [23]. Given that ED and AD share the proposed etiology of being “stress-induced”, it is plausible that there is a diagnostic overlap between the constructs where AD might be conceptualized as a prodromal phase to ED. Importantly, however, given the exploratory nature of the subgroup analyses in the present study, results should be interpreted with caution.

The relationship between subjective symptoms and self-reported memory impairment with cognitive test results was also examined in this study. Consistent with results from previous studies [13, 18, 20, 21], the present study found weak or no correlations between subjective complaints and objective cognitive functioning. For patients with functional neurological disorders, fibromyalgia, and chronic fatigue syndrome, it has been suggested that subjective cognitive impairment may be more closely connected to perfectionistic traits, heightened threat perception, excessive self-monitoring, awareness of cognitive failures, and negative self-thoughts rather than objective cognitive functioning as measured with cognitive tests [22, 38]. Following this line of reasoning, the large effect sizes of subjective cognitive impairments usually seen in these patients [38, 39], as in patients with ED, could partly be the result of an attentional bias, exacerbated by catastrophic interpretations of cognitive failures and unrealistic self-expectations, and not of objective cognitive impairment. Therefore, to ensure that interventions are effective in improving cognitive functioning in patients with stress-related mental disorders, it is crucial to have a clear understanding of the underlying mechanisms of both subjective and objective impairment. Future research should focus on developing comprehensive theoretical models that can explain the relationship between these constructs, guiding the development of more targeted and effective interventions for patients with stress-related mental disorders.

### Clinical implications

To contextualize the results, it is important to note that although patients as a group performed worse than the healthy reference group on all cognitive measures, none of the tests showed results on a group level that would qualify as a neurocognitive impairment according to the DSM-5. APA recommendations define less-than-typical performance if below 1 SD, suspected impairment if below -1.5 SD, and pathological or atypical impairment if below 2 SD from expected score in a healthy population [40]. Given these guidelines and the results of the present study, objective cognitive tests in patients with stress-related mental disorders may have limited clinical utility. Further, it is important to note that although patients with AD and ED report subjective cognitive impairment and, in the present study, were found to perform worse than a healthy reference group on objective cognitive tests, similar impairments have also been reported in other psychiatric [41, 42], neuropsychiatric [43] and somatic conditions [38, 39, 44]. For instance, patients with depressive disorders have been found to exhibit cognitive impairments in attention and processing speed, executive functioning, memory, and psychomotor speed with small to moderate effect sizes using objective cognitive tests compared to healthy controls [41]. For patients diagnosed with chronic fatigue syndrome, reports of high levels of subjective impairment are common [39, 45] but findings on objective impairments are heterogenous [22]. A similar picture emerges for patients with fibromyalgia where one study found no difference in objective test performance compared with controls, but high levels of subjective impairment [38]. In another study, fibromyalgia patients showed high levels of subjective impairment and executive dysfunction compared to controls, though most differences disappeared when adjusting for depression and anxiety [44]. In summary, the experience of cognitive difficulties is not a unique characteristic of stress-related mental disorders. Given results from the present study and those from studies of other diagnostic constructs with similar clinical pictures, it is unlikely that current objective cognitive testing is useful for assessment and diagnostic purposes, i.e., for discriminating AD from ED or stress-related mental disorders from a range of other psychiatric and somatic disorders. Rather, future research avenues might include investigating whether cognitive function could predict treatment outcome as has been suggested for patients with depression [46], obsessive compulsive disorder [47] and in other psychiatric disorders [48], and whether specific treatment interventions may affect subjective cognitive impairment over time. Further, at present, it is not known if subjective difficulties represent individual changes that debuts with the disorder, or if they represent pre-morbid vulnerability factors. Such investigations are facilitated by digitized testing procedures as used in the present study, enabling large-scale and cost-effective testing.

### Strengths and limitations

The main strengths of the present study include the large sample size, the well-defined sample (using existing diagnostic constructs as opposed to, e.g., burnout) that facilitates transparency and generalizability of findings, and the standard automatized administration procedure including the use of validated cognitive tests. Although it remains to be empirically tested, the remote testing procedure could possibly have allowed for a more naturalistic testing environment, thus increasing ecological validity of results. However, we cannot exclude that it may have simultaneously introduced potential sources of bias such as unexpected disturbances during testing. Although comparative validity between digitized and paper and pen cognitive testing procedures have been shown [49], remote cognitive testing is still in its infancy and more research is needed to fully understand the pros and cons as compared to traditional procedures [50]. Lastly, the study protocol including a detailed statistical analysis plan and code was published on Open Science Framework, which is important for research transparency and replicability.

A limitation to the present study was that the test battery used was, for clinical reasons, designed to take at most 20 min to complete. It has previously been argued that shorter testing sessions may not allow for the full effects of fatigue to impact the patients' cognitive performance, potentially underestimating actual cognitive impairment and likely reducing the possibility to properly estimate effect sizes for the different domains [51]. The cross-sectional design in turn also did not enable analysis of whether the stress-related mental disorders developed first and resulted in cognitive impairments, or whether cognitive impairments are a predisposing vulnerability that increases the risk of developing a stress-related mental disorder. Additionally, it was a limitation to the study that the test battery used did not contain any measure to assess malingering or motivation, i.e., performance validity. Lastly, it should be noted that the data collection was conducted during the Covid-19 pandemic, and it cannot be excluded that this affected the well-being, or possibly cognitive performance of the participants. Because neither indices of well-being nor stress have been indicated deteriorate uniformly across groups or countries [52], a strong effect of the pandemic on the observed results is unlikely.

## Conclusions

The present study adds to previous clinical observations and research findings indicative of small to moderate objective cognitive impairments in patients diagnosed with stress-related mental disorders. For the first time, objective cognitive functioning in different stress-related mental disorders (i.e., AD and ED) were compared, with results suggesting that even though patients with ED appear to have more wide-spread cognitive difficulties than patients with AD when compared with healthy norms, no significant differences in cognitive functioning were found when the diagnostic groups were compared with each other. This, and the fact that similar cognitive impairments have been found across a range of psychiatric and somatic conditions, suggests that cognitive impairment might be conceptualized as a dimensional transdiagnostic factor rather than a diagnosis-specific phenomena. Further exploration into mechanisms of cognitive functioning in different populations is merited to enable development of theoretical models that may explain the weak correlation between self-reported cognitive impairments and objective measures.

### Supplementary Information

**Additional file 1.** 

## Data Availability

The data that was analyzed and or generated is not openly available due to Swedish law (the Swedish Ethical Review Act: 2003:460) but can be made available by the authors on reasonable request. For such requests, please contact EL.
