# Supplementary material for: Objective cognitive functioning in patients with stress-related disorders: a cross-sectional study using remote digital cognitive testing
Source: BMC Psychiatry. 2023 Aug 7;23:565. doi: 10.1186/s12888-023-05048-5 (PMC10405463; doi:10.1186/s12888-023-05048-5)
Supplement: Supplementary file 1 — Additional file 1. [file 12888_2023_5048_MOESM1_ESM.pdf]

# SUPPLEMENTARY MATERIAL

# Objective Cognitive Functioning in Patients with Stress-Related Disorders:

*A Cross-Sectional Study Using Remote Digital Cognitive Testing*

Ludwig Franke Föyen[1,3,5*], Victoria Sennerstam[1,4,5], Evelina Kontio[1,5], Mats Lekander[1,3,4], Erik Hedman-Lagerlöf[1,4,5], and Elin Lindsäter[1,2,4,5]

[1] Division of Psychology, Department of Clinical Neuroscience, Karolinska Institutet, Stockholm, Sweden.

[2] Center for Psychiatry Research, Department of Clinical Neuroscience, Karolinska Institutet, & Stockholm Health Care Services, Region Stockholm, Sweden.

[3] Stress Research Institute, Department of Psychology, Stockholm University, Stockholm, Sweden.

[4] Osher Center for Integrative Health, Department of Clinical Neuroscience, Karolinska Institutet, Stockholm, Sweden.

[5] Gustavsberg University Primary Care Center, Stockholm Health Care Services, Region Stockholm, Sweden

[*] Corresponding author: Ludwig Franke Föyen, Department of Clinical Neuroscience, Karolinska Institutet, Gustavsberg University Primary Care Center, Odelsbergs väg 19, SE-134 40 Gustavsberg, Sweden. E-mail: Ludwig.franke-foyen@su.se

| **Contents** | **Pages** |
|---|---|
| Table S1. Diagnostic Criteria for exhaustion disorder (ED) (F43.8 A) published by the National Board of Health and Welfare in Sweden | 3 |
| 6 – Questionnaire of Everyday Memory Problems (Swedish version) | 4 |
| Example of how a virtual control was generated | 6 |
| Sensitivity analysis | 7 |
| Table S2. Sensitivity analysis of differences between patients with stress-related mental disorders compared to healthy normative group. Outliers +-3 z scores excluded. | 8 |
| Table S3. One-way ANOVA for each cognitive test comparing the diagnostic groups AD and ED with the control group | 9 |
| Figure S1. Heatmap showing the relationship between test scores and subjective burnout symptoms and memory impairment | 10 |
| List of abbreviations | 11 |

**Table S1**. *Diagnostic Criteria for exhaustion disorder (ED) (F43.8 A) published by the National Board of Health and Welfare in Sweden*[a]

A. Physical and mental symptoms of exhaustion during at least 2 weeks. The symptoms have developed in response to one or more identifiable stressors, which have been present for at least 6 months.

B. Markedly reduced mental energy, manifested by reduced initiative, lack of endurance, or increased time needed for recovery after mental efforts.

C. At least four of the following symptoms have been present most of the day, nearly every day, during the same 2-week period:

1. Persistent complaints of impaired memory and concentration.
2. Markedly reduced capacity to tolerate demands or to perform under time pressure.
3. Emotional instability or irritability.
4. Insomnia or hypersomnia.
5. Persistent complaints of physical fatigue and lack of endurance.
6. Physical symptoms such as muscular pain, chest pain, palpitations, gastrointestinal problems, vertigo, or increased sensitivity to sounds.

D. The symptoms cause clinically significant distress or impairment in social, occupational, or other important areas of functioning.

E. The symptoms are not due to the direct physiological effects of a substance (e.g., abuse of a drug or medication) or a general medical condition (e.g., hypothyroidism, diabetes, infectious disease).

F. If criteria for major depression, dysthymia, or generalized anxiety disorder are met simultaneously, exhaustion disorder is set as an additional specification to any such diagnosis.

*a All criteria with capital letters must be met to set the diagnosis.*

**6 – Questionnaire of Everyday Memory Problems (Swedish version)**

1. Tycker någon i din närhet (familj, vänner) att du har dåligt minne?

    Aldrig

    Sällan

    Ibland

    Ofta

    Vanligtvis

2. Hur tycker du att ditt minne är idag, jämfört med hur det var innan dina stressrelaterade besvär debuterade?

    Mycket bättre

    Något bättre

    Lika

    Något sämre

    Mycket sämre

3. Hur tycker du att ditt minne är idag, jämfört med hur det var när du besvärades som mest av dina stressrelaterade symtom?

    Mycket bättre

    Något bättre

    Lika

    Något sämre

    Mycket sämre

4. Hur upplever du ditt minne i jämförelse med andra personer i din ålder?

    Mycket bättre

    Något bättre

    Lika

    Något sämre

    Mycket sämre

5. Glömmer du tider om du inte påminns av någon annan eller skriver upp det i en kalender eller dagbok?

    Mycket ofta

    Ganska ofta

    Ibland

    Sällan

    Aldrig

6. Händer det att du inte kommer ihåg saker som har hänt under de senaste dagarna?

    Mycket ofta

    Ganska ofta

    Ibland

    Sällan

    Aldrig

**Example of how a virtual control was generated**

For the first exploratory analysis, to compare the diagnostic groups separately with the normative group, a virtual control was generated for each patient by taking the expected cognitive test results given the model, and its standard deviation, and generating a result from a normal distribution with the expected score as mean. For example: 36-year-old woman, with 14 years of education using a mouse had a predicted score of 52 on SDMT. Using this information, together with the model standard deviation, in this case 6.615, a virtual control could be generated by randomizing a score from a normal distribution with the predicted score of 52 as mean, and the model SD as the standard deviation. Creating a virtual normative group was done as an alternative to conducting multiple one-sample *t*-tests (7x3) for each test and diagnostic group.

**Sensitivity analysis**

A post-hoc sensitivity analysis was performed with the same statistical plan but excluding all outliers as indicated by participant score +- 3 *z*-scores indicating 3 standard deviations above or below expected score. This resulted in 5 additional participants being excluded from CERAD learning, 2 from CERAD recall, 2 from Corsi, 2 from SDMT, 6 from Stroop index, and 9 from Stroop interference. In this analysis, only CERAD recall and Corsi was considered non-normal and thus Wilcoxon signed-rank test was used for these tests. In comparing patients with stress-related mental disorders to the healthy normative group using this analysis, we found worse performance in all but one test, Stroop interference, see Table S2.

**Table S2.** *Sensitivity analysis of differences between patients with stress-related mental disorders compared to healthy normative group. Outliers +-3 z scores excluded.*

| Cognitive test | *Mean* (*SD*) [1] | Test statistic[2] | *df* | *p-value*[3] | Effect size[2] | 95% CI |
|---|---|---|---|---|---|---|
| **Attention and processing speed** | | | | | | |
| SDMT | -.42 (.89) | -7.62 | 263 | **<.001** | -.47 | [-.60, -.35] |
| **Executive functions** | | | | | | |
| FAS | -.11 (.82) | -2.03 | 243 | **.022** | -.13 | [-.26, 0] |
| Stroop index | -.54 (1.02) | -8.59 | 259 | **<.001** | -.53 | [-.65, -.47] |
| Stroop interference | -.1 (1.01) | -1.56 | 256 | .060 | -.1 | [-.28, -.01] |
| **Memory** | | | | | | |
| CERAD learning | -.25 (.98) | -4.02 | 239 | **<.001** | -.26 | [-.44, -.18] |
| CERAD recall | -.47 (.87) | 6983 | 242 | **<.001** | -.53 | [-.63, -.47] |
| Corsi | -.18 (.86) | 12174 | 263 | **<.001** | -.3 | [-.43, -.19] |

[1] *Mean* signifies the mean of *z*-scores in the patient group.

[2] For CERAD learning and recall, Corsi, and Stroop interference and index, Test statistic is the value of the *W*-statistic and effect size is given by the matched rank biserial correlation. For FAS and SDMT, the Test statistic is the t-statistic, and the effect size is given by Cohen's d.

[3] For the Student *t-test*. the alternative hypothesis specifies that the mean is less than 0. For the Wilcoxon test the alternative hypothesis specifies that the median is less than 0.

*Abbreviations.* CERAD = Consortium to Establish a Registry for Alzheimer’s Disease, SDMT = Symbol Digit Matching Task

**Table S3.** *One-way ANOVA for each cognitive test comparing the diagnostic groups AD and ED with the control group*

| Cognitive test | *ddof1* | *ddof2* | *F* | *p* | *np2* |
|---|---|---|---|---|---|
| **Attention and processing speed** | | | | | |
| SDMT | 2 | 529 | 11.26 | **<.001** | .04 |
| **Executive functions** | | | | | |
| Stroop index | 2 | 529 | 26.91 | **<.001** | .09 |
| Stroop interference | 2 | 529 | 3.97 | **0.02** | .01 |
| **Memory** | | | | | |
| CERAD learning | 2 | 487 | 5.49 | **0.004** | .02 |
| CERAD recall | 2 | 487 | 16.82 | **<.001** | .06 |
| Corsi | 2 | 529 | 3.8 | **0.023** | .01 |

*Abbreviations.* CERAD = Consortium to Establish a Registry for Alzheimer's Disease, SDMT = Symbol Digit Matching Task

**Figure S1**. *Heatmap showing the relationship between test scores and subjective burnout symptoms and memory impairment*

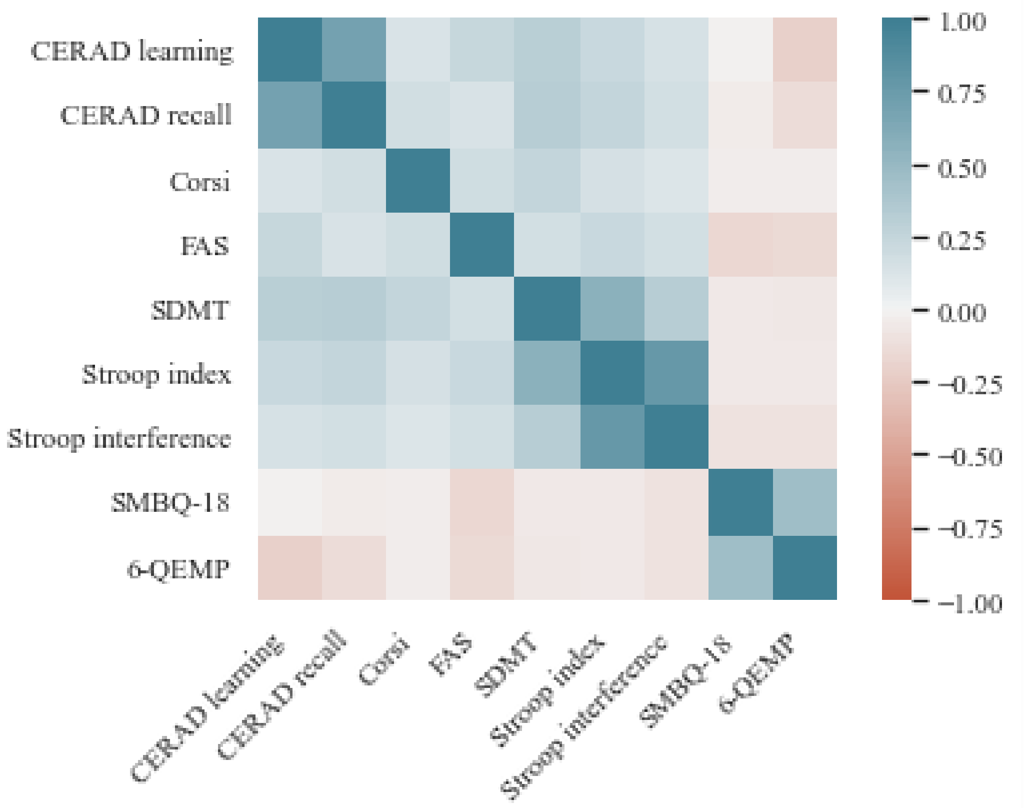


*Abbreviations.* CERAD, Consortium to Establish a Registry for Alzheimer’s Disease; SDMT, Symbol Digit Matching Task. SMBQ, Shirom-Melamed Burnout Questionnaire. 6-QEMP, 6 – Questionnaire of Everyday Memory Problems

## List of abbreviations

| | |
|---|---|
| 6-QEMP | Six-item questionnaire of Everyday Memory Problems |
| AD | Adjustment disorder |
| ANOVA | Analysis of variance |
| CERAD | Consortium to Establish a Registry for Alzheimer's Disease |
| DSM | Diagnostic and Statistical Manual of Mental Disorders |
| ED | Exhaustion disorder |
| ICD | International classification of diseases |
| RCT | Randomized controlled trial |
| SDMT | Symbol Digit Matching Task |
| SMBQ | Shirom-Melamed Burnout Questionnaire |
